# Uterine WNTS modulates fibronectin binding activity required for blastocyst attachment through the WNT/CA^2+^ signaling pathway in mice

**DOI:** 10.1186/s12958-023-01135-0

**Published:** 2023-09-15

**Authors:** Yuefei Lou, Laurie Pinel, Daniel Dufort

**Affiliations:** 1 Department of Obstetrics and Gynecology, McGill University, Montreal, QC H4A 3J1 Canada; 2 Child Health and Human Development Program, Montreal, QC H4A 3J1 Canada; 3 Research Institute of the McGill University Health Centre, Montreal, QC H4A 3J1 Canada; 4 Present Address: Department of Critical Care Medicine, Cumming School of Medicine, University of Calgary, Calgary, AB T2N 1N4 Canada

**Keywords:** Wnt signaling, Embryo implantation, Integrins, Fibronectin binding, Mouse blastocyst, Trophectoderm, Wnt/Ca^2+^, Non-canonical wnt signaling

## Abstract

Adhesion of the implanting blastocyst involves the interaction between integrin proteins expressed by trophoblast cells and components present in the basement membrane of the endometrial luminal epithelium. Although several factors regulating integrins and their adhesion to fibronectin are already known, we showed that Wnt signaling is involved in the regulation of blastocyst adhesion through the trafficking of integrins expressed by trophoblast cells. Localization of Itgα5β1 by immunofluorescence and FN-binding assays were conducted on peri-implantation blastocysts treated with either Wnt5a or Wnt7a proteins. Both Wnt5a and Wnt7a induced a translocation of Itgα5β1 at the surface of the blastocyst and an increase in FN-binding activity. We further demonstrated that uterine fluid is capable of inducing integrin translocation and this activity can be specifically inhibited by the Wnt inhibitor sFRP2. To identify the Wnt signaling pathway involved in this activity, blastocysts were incubated with inhibitors of either p38MAPK, PI3K pathway or CamKII prior to the addition of Wnts. Whereas inhibition of p38MAPK and PI3K had not effect, inhibition of CamKII reduced FN-binding activity induced by Wnts. Finally, we demonstrated that inhibition of Wnts by sFRP2 reduced the binding efficiency of the blastocyst to uterine epithelial cells. Our findings provide new insight into the mechanism that regulates integrin trafficking and FN-binding activity and identifies Wnts as a key player in blastocyst attachment to the uterine epithelium.

## Introduction

Embryo implantation is a critical step of pregnancy that requires extensive communications between the blastocyst and the maternal endometrium. Indeed, both the hatched blastocyst floating in the uterine lumen and the luminal epithelial cells of the receptive endometrium need to be synchronized for implantation to occur [1, 2]. This communication between the blastocyst and the uterus involves signalling pathways, growth factors and important hormonal regulation and implicates both intrinsic and extrinsic factors that affect the blastocyst and the uterus [3]. Failure in embryo implantation is not only a problem after natural conception but also a major limiting factor in assisted reproduction [4]. It can be attributed to several reasons classified in three main categories: embryonic (such as non-competent or genetically abnormal blastocyst), uterine (e.g. non-receptive endometrium) or a problem in maternal-fetal communication [5]. There have been significant advances in recent years in identifying factors and criteria involved in the development of healthy embryos and rendering the uterus in an implantation competent state. However, very little is known on the maternal-fetal communication required for coordinating successful embryo implantation. Thus, it is crucial to decipher and identify the factors regulating this complex and crucial maternal-fetal crosstalk.

The implantation process can be subdivided into multiple steps including apposition, adhesion, and invasion of the blastocyst into the maternal uterine tissue. The apposition step involves a weak interaction between the pinopods of the endometrium and the trophectoderm cells of the blastocyst and occurs in the mouse towards the end of day 4 of the pregnancy (vaginal plug = day 1) [6]. The adhesion step predominantly involves integrin proteins expressed by trophoblast cells. Integrins are heterodimeric transmembrane glycoproteins that bind to the extra-cellular matrix (ECM) components present in the basal membrane of the endometrial epithelium such is composed of fibronectin, vitronectin, entactin and collagen [7–11]. Several integrins are expressed by the trophoblast cells of blastocysts and both their expression and localization changes during the peri implantation period [12, 13]. In the early blastocyst, integrin αVβ3 is expressed at the apical surface of non-adhesive trophoblast cells whereas Itgα5β1 and αIIb subunits are localized intracellularly or at the basal cell surface. In response to uterine factors, the blastocyst becomes adhesion-competent and Itgα5β1 migrates to the apical plasma membrane of trophoblast cells and its binding with fibronectin allows the trafficking of αIIb subunits to the apical surface [13–15]. Use of antibodies against αv, α5, β1 and β3 integrin subunits decreased fibronectin (FN)-binding activity or FN-mediated outgrowth confirming their important role in the implantation of the blastocyst [7, 9].

Numerous studies have investigated the regulatory mechanisms of the FN-binding activity in the blastocyst attributed to integrin proteins [16, 17]. Early studies demonstrated that the regulation of integrin during peri-implantation is not through gene expression or protein synthesis but mainly by integrin trafficking [7, 18]. Later several in vitro studies demonstrated that integrin protein trafficking is regulated by multiple bioactive factors including calcitonin, lysophosphatidic acid and HB-EGF [16]. Blastocysts treated in vitro with calcitonin; a hormone secreted just before implantation showed accelerated outgrowth with an early peak of FN-binding activity after 48 h of culture which was associated with an early translocation of Itgα5β1 to the surface of blastocysts [19]. Exposure of blastocysts to a cannabinoid agonist leaded to the same increase of FN-binding activity after 48 h of culture [20]. HB-EGF was also described to accelerate trophoblast development to an adhesion-competent stage in culture by triggering an early presence of Itgα5β1 on the surface of blastocyst [21]. As the migration of integrins to the surface of mural trophectoderm is a crucial phenomenon allowing the adhesion of the blastocyst to the endometrium, it is important to further identify the factors regulating this trafficking. Although several factors have been shown to accelerate trophoblast development to an adhesion-competent stage in vitro, the in vivo signal involved in modulating integrin trafficking required for the blastocyst to reach an adhesion-competent state is still unknown.

We and others have previously characterized Wnt gene expression in the mouse uterus and in the blastocyst during the peri-implantation period and demonstrated that the Wnt signalling pathway is active in the blastocyst as well as in areas of the uterine endometrium directly apposed to the blastocysts at the time of implantation [22–25]. Moreover, studies demonstrated that inhibition of this pathway altered embryo implantation in the mouse [22, 26]. Transferring blastocysts into recipient females with the sFRP2 protein, which prevents the ligation of Wnts proteins with their receptors, induced a decrease in implantation frequency compared with blastocysts transferred alone [22]. Silencing of Wnt-β-catenin signaling in embryo via an adenoviral vector also induced a lower implantation rate in treated females [26]. In addition, the correlation between the Wnt signaling pathway and the cell adhesion process has already been described [27–29]. For instance, silencing of WNT4 using siRNA in the HTR-8/SVneo trophoblast cell lines induced a decrease in ITGA2 and ITGAV expression [28]. Therefore, we hypothesized that the uterine Wnt signalling pathway may be involved in the regulation of trophoblast adhesion through the trafficking of integrins expressed by the trophectoderm cells of the blastocyst.

In our study, the effect of two Wnts proteins were evaluated; Wnt5a and Wnt7a since both are expressed in the uterus prior to embryo implantation [23]. We demonstrate that addition of either Wnt5a or Wnt7a into the blastocysts culture media causes relocalization of Itgα5β1 to the apical surface of mural trophectoderm resulting in increased fibronectin binding activity. Furthermore, we show that exposure of newly hatched blastocysts to uterine fluid or a short in vivo uterine exposure, resulted in increased fibronectin binding activity in the mural trophectoderm. This activity, both in vivo and in uterine fluid, can be inhibited by the addition of the Wnt inhibitor sFRP2 and can be rescued by the addition of excess Wnts. Finally, we demonstrate that integrin translocation by Wnts is modulated through the Wnt calcium intracellular signaling pathway (Wnt/Ca^2+^). Our results demonstrate a new role for Wnt signalling in the acquisition of an adhesion-competent blastocyst state prior to implantation.

## Materials and methods

### Collection and culture of embryos

Experimental protocols in this study were in accordance with regulations established by the Canadian Council on Animal Care and were reviewed by the Animal Care Committee of the McGill University Health Centre, #MUHC 5261. Embryos were obtained as previously described [30]. Briefly, CD-1 females (Charles River, Montreal, QC Canada) were superovulated by injection of 5 IU of PMSG (Sigma-Aldrich, Mississauga, ON) followed 44 h later by 5 IU of hCG (Sigma-Aldrich). Female mice were mated individually with CD-1 males overnight. Mating was indicated by the presence of a vaginal plug the following morning (designated as day 0.5). Embryos were collected on day 3.5 (3.5dpc) by flushing the uterine horns with M2 medium (Sigma-Aldrich) and cultured in 10-μl droplets of KSOM medium (Sigma-Aldrich) under mineral oil (Sigma-Aldrich) at 37° C in 5% CO_2_ for 48 h until hatched.

### Preparation of soluble Wnt proteins

Parental L cells, Wnt5a cells, and Wnt7a cells were generated as previously described [22]. Wnt-expressing cells were grown in DMEM (GIBCO, Burlington, ON, Canada), 10% heat-inactivated FBS (Wisent Bioproducts, Saint-Bruno, QC, Canada)), 1 mM L-glutamine (GIBCO) and selected with 5 μg/ml Puromycin (Sigma-Aldrich). Parental L cells, Wnt5a cells, and Wnt7a cells were seeded to 70% confluence, and the condition media (CM) containing active Wnt5a, Wnt7a, and CM from control L cells were collected 3 days later. Cell debris were removed from the CM by centrifugation at 3000 rpm for 10 min and filtered through a 0.22 μm syringe filter. The conditioned media was kept at 4 °C for no more than 4 weeks prior to use.

### Preparation of embryos for in vitro and in vivo assay

Embryos were treated in KSOM medium (Sigma-Aldrich) with 10% Wnt5a CM or Wnt7a CM or control CM for 48 h before immunofluorescence. After treatment embryos were washed and cultured in KSOM medium (Sigma-Aldrich) for one additional hour prior to the FN-binding assay. Where indicated, the embryos were pre-treated with 5 μm KN93, 1 μm Wortmanmin, or 5 μm SB202190 (all from Sigma-Aldrich) for 30 min before adding Wnt CM.

For in vivo experiments, hatched blastocysts either alone, or with 500ng of sFRP2 (R&D Systems, Minneapolis, MN), or with sFRP2 plus 10% Wnt7a CM, were transferred into the uterine horn of pseudopregnant females on day 4. The embryos were flushed out from the uterine horn 5 min after injection and then cultured in KSOM medium (Sigma-Aldrich) for one additional hour before the FN-binding assay.

### FN-binding assay

FN-binding activity was carried out as previously described [7]. FN-120 (Bioscience Research Reagents (formerly Chemicon)) - coated fluorescent-green, 1.0 mm, polystyrene microspheres (Bang’s Laboratories, Carmel, IN) were used for assaying the FN-binding activity of the treated blastocysts. The FN-binding assay was carried out by incubating the embryo in 10 μl drops of FN120 coated fluorescent microspheres for 30 min at 4 ^°^C.

Following incubation, the embryos were washed in cold PBS/BSA (Milipore, Sigma-Aldrich) six times and then fixed in 4% paraformaldehyde (Sigma-Aldrich) for 30 min at room temperature. The embryos were washed and nuclear stained with 0.5ug/ml propidium iodide (PI) (Sigma-Aldrich) in PBS/BSA (Milipore, Sigma-Aldrich) for 30 min at room temperature. Embryo-bound microspheres (green) and nuclei staining (red) were viewed on a Zeiss 510 confocal scanning laser microscope (Thornwood, NY) using excitation wavelengths of 488 and 543 nm respectively. Images shown in results were representative of at least 15 embryos that produced similar staining patterns. All confocal images were taken under identical conditions.

The fluorescence intensity of bound microspheres was measured using MetaMoph version 6.1 software. The mean fluorescence (green level) intensity of ten to fourteen equally spaced relative optical density values were averaged along the in-focus edge of each digitized image over 180^o^ of embryonic or abembryonic pole using a small square tool projected by the MetaMoph software system. Average relative fluorescent intensity was pooled from at least three to five embryos.

### Immunofluorescence and confocal microscopy

Blastocysts were fixed at room temperature for 15 min in fresh 4% PFA (Sigma-Aldrich) in PBS, permeabilized for 15 min with 0.1% Triton X-100 (ThermoFisher) in PBS, blocked in PBS containing 3% BSA. Six drops of PBS containing 10 mg/ml BSA (PBS/BSA) were used to rinse embryos after all incubations throughout the procedure. The embryos were then incubated in anti-integrin α5β1 primary antibody (MAB2514, Milipore, Sigma-Aldrich) overnight at 4 °C and in Cy2-conjugated secondary antibody (Jackson ImmunoResearch, West Grove, PA) for 1 h at room temperature. For non-permeable staining, embryos were incubated with anti-integrin α5β1 primary antibody in KSOM medium (Sigma-Aldrich) at 37 °C for 1 h and then washed six times with M2 medium (Sigma-Aldrich). After washing embryos were incubated in the Cy2 secondary antibody (Jackson ImmunoResearch) at 37 °C for 45 min. Embryo were fixed in 4% PFA (Sigma-Aldrich) in PBS. The embryos were incubated in PBS containing nuclear staining PI (Sigma-Aldrich) and mounted on glass slides. The embryos were viewed in a Zeiss (Thornwood, NY) 510 confocal scanning laser microscope.

### Isolation of uterine epithelial cells and co-culture with blastocysts

3.5 dpc mice were euthanized by isofluorane inhalation and cervical dislocation. Uteri were removed from the mouse, cleared from fat tissues, and flushed with M2 medium (Sigma-Aldrich). Uteri were digested in 0.25% trypsin/EDTA (Wisent Bioproducts) at 4 °C for 60 min and at room temperature for 30 min. Luminal epithelial sheets were flushed out with Hank’s Mg^++^ and Ca^++^ free (ThermoFisher), collected and centrifuged at 2000 rpm for 5 min at room temperature. Cells pellets were resuspended into Hank’s Mg^++^ and Ca^++^ free (ThermoFisher) with trypsin (200 μg/ml Sigma-Aldrich) and incubated at room temperature for 5 min on the shaker. Cells sheets were broken by pipetting up and down several times. Trypsin inhibitor (300 μg/ml; Milipore, Sigma-Aldrich) and DNAse (100 μg/ml; Sigma-Aldrich) were added to the mix and incubated for 2 min at room temperature before the addition of 4% FBS (Wisent Bioproducts). Cells were centrifuged at 2000 rpm for 5 min at room temperature and resuspended in 0.8% NH4CL in 0.1mM EDTA for 1 min before centrifugation step at 2000 rpm for 5 min at room temperature. Cells were washed three times with Hank’s Mg^++^ and Ca^++^ free (ThermoFisher), before being counted. Cells were resuspended in DMEM/F12 (Wisent Bioproducts) with 10% FBS (Wisent Bioproducts) and plated into a glass-bottom dish coated with 5 μg/cm^2^ collagen (Corning, Lowell, MA) and cultured at 37 °C in a 5% CO_2_ incubator for 24 to 48 h. Meanwhile, blastocysts were cultured in KSOM (Sigma-Aldrich) until they hatched (around 24 to 48 h). Medium from epithelial cells was removed and replaced by KSOM alone, or with 500ng of sFRP2 (R&D Systems), or with sFRP2 plus 10% Wnt7a CM for 1 h at 37° C. After incubation, hatched blastocysts were added to the cells and incubated for 2 h at 37° C. Percentage of attachment of the blastocyst were evaluated by shaking the cultures and counting the non attached and attached blastocysts under a light microscope.

### Statistics

Data are presented as the mean ± standard error of the mean of independent samples. Statistical analyses were done using GraphPad Prism (version 9; GraphPad Software Inc., San Diego, CA). Statistical analysis comparing experimental groups was performed using ANOVA test with Holm-Sidak or Dunnett’s multiple comparison test. P-values less than 0.05 were considered statistically significant. In the figures, data were not significant unless otherwise indicated.

## Results

### Wnt proteins increased Itgα5β1 expression at the surface of the mural trophectoderm

**Fig. 1:**
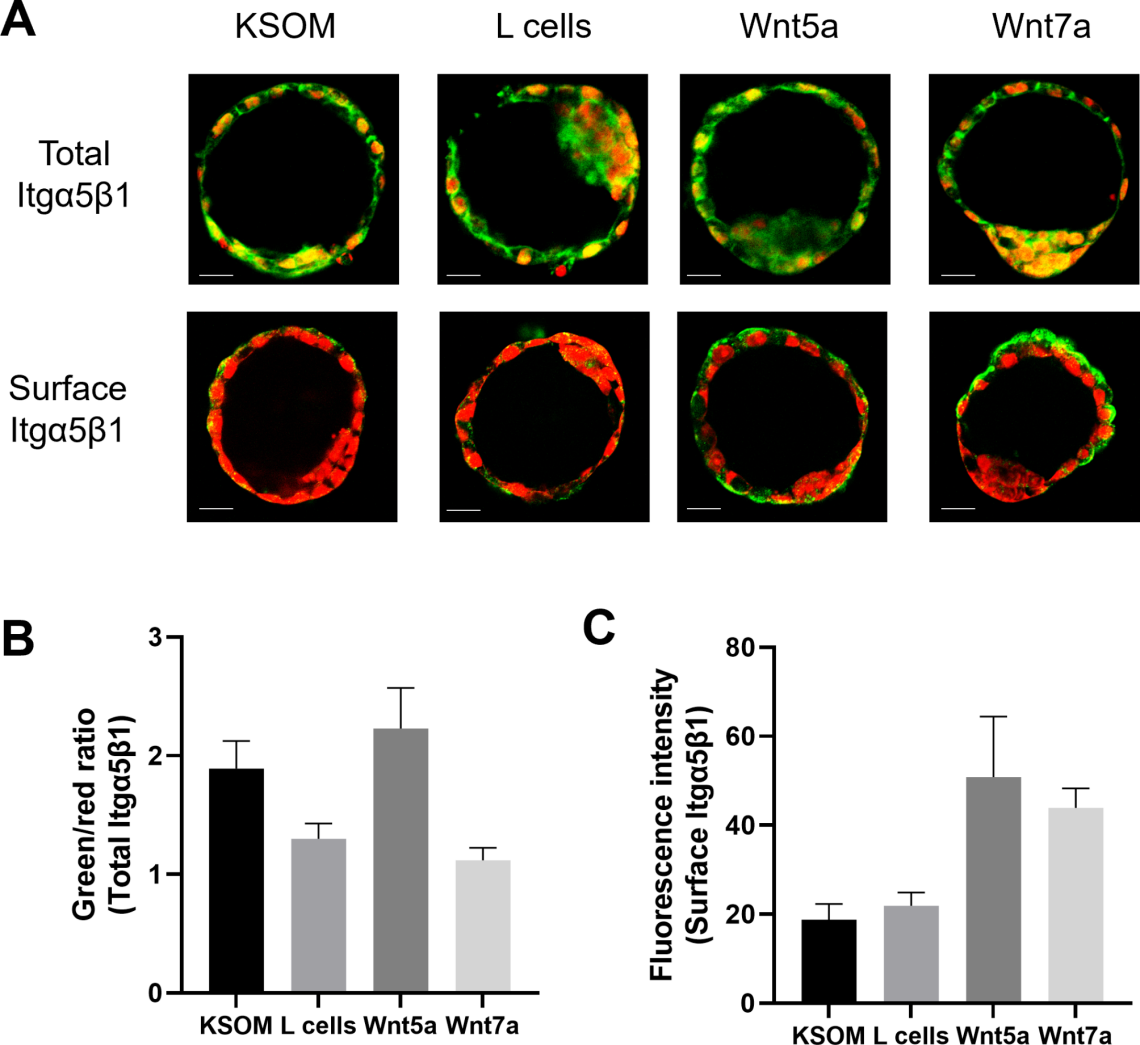
Expression of Itgα5β1 in peri-implantation blastocysts treated with Wnt proteins. (**A**) Microscopy images of 3.5 dpc blastocysts cultured in KSOM alone or with control CM (L cells), Wnt5a CM and Wnt7a CM and stained with (total Itgα5β1) or without (surface Itgα5β1) permeabilization with anti-Itgα5β1 antibody (green). Nuclei were stained with iodure propidium (red). (**B**) The ratio between the green signal (Itgα5β1) and red signal (iodure propidium) was used to evaluate the total Itgα5β1 expression in 3.5 dpc blastocysts cultured for 48 h in KSOM alone, control CM, Wnt5a CM and Wnt7a CM and stained with permeabilization. (**C**) The fluorescent intensity of the green ratio (Itgα5β1) was used to evaluate the surface Itgα5β1 expression in 3.5 dpc blastocysts cultured for 48 h in KSOM alone, control CM, Wnt5a CM and Wnt7a CM and stained without permeabilization. * ANOVA test, P < 0.05

We chose to focus on Itgα5β1 as this integrin is responsible for most of the FN-binding activity and previous investigations have demonstrated a correlation between FN-binding activity and expression of the Itgα5β1 on the apical surface of trophoblast cells [7, 14]. To evaluate the role of Wnt proteins on the expression and localization of Itgα5β1, we conducted immunofluorescence staining with anti-Itgα5β1 antibody on 3.5dpc blastocysts cultured for 48 h in KSOM alone or in KSOM with the addition of either 10% control L-cells conditioned media (CM), 10% Wnt5a or Wnt7a CM. To evaluate changes in total expression of Itgα5β1 in response to Wnts, blastocysts were permeabilized prior to staining. Itgα5β1 showed homogeneous and abundant staining through all the cells of the blastocyst (Fig. 1A). However, staining without permeabilization to only visualize the Itgα5β1 expression on the apical surface of the trophectoderm, showed a weak signal on the surface of the mural trophectoderm cells in blastocysts incubated with KSOM alone or with control L cell CM. Interestingly, stronger staining was observed in the mural trophectoderm cells of blastocysts treated with 10% Wnt5a CM and 10% Wnt7a CM (Fig. 1A). The ratio between the green signal (Itgα5β1) and red signal (propidium iodide stained DNA) was used to evaluate the total Itgα5β1 expression in blastocysts. This ratio showed no significant change in total Itgα5β1 in response to the addition of Wnts (Fig. 1B) (ratio of 1.89 ± 0.32 for KSOM alone, 1.29 ± 0.18 for L cell control CM, 2.21 ± 0.50 for 10% Wnt5a CM and 1.11 ± 0.14 for 10% Wnt7a CM) whereas the fluorescent intensity on the apical surface of Itgα5β1 tended towards an increase after treatment with 10% Wnt5a and 10% Wnt7a CM (Fig. 1C) (ratio of 18.73 ± 4.99 for KSOM alone, 21.91 ± 5.15 for L cell control CM, 50.83 ± 23.51 for 10% Wnt5a CM and 43.87 ± 7.71 for 10% Wnt7a CM). These findings suggested that exposure of blastocysts to Wnt5a or Wnt7a proteins accelerated migration of the Itgα5β1 to the apical surface of blastocysts.

### Wnt proteins increased the FN-binding activity of the mural trophectoderm

**Fig. 2:**
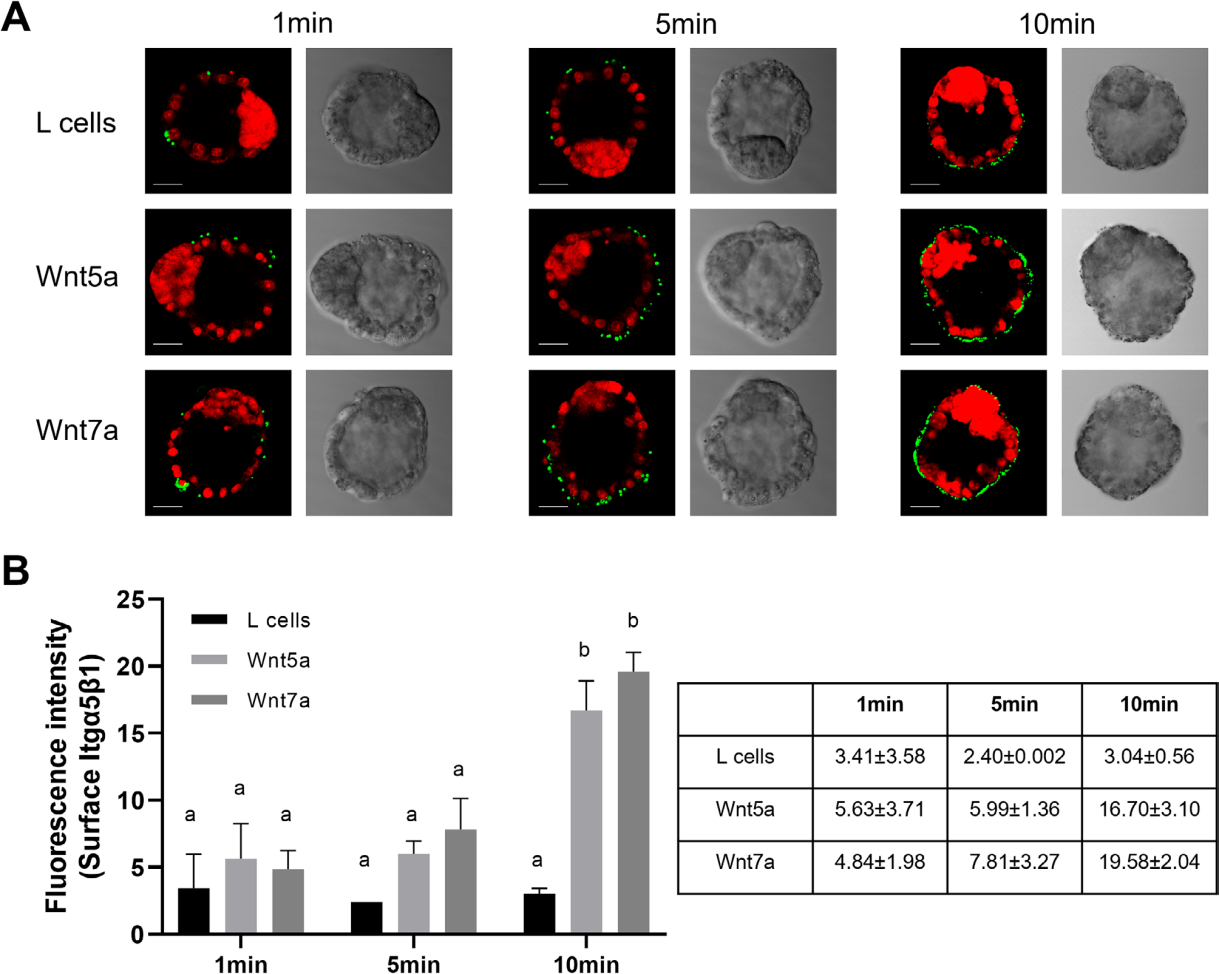
FN-Binding assays of peri-implantation blastocysts treated with Wnt proteins. (**A**) Microscopy images of FN-binding assays realized on 3.5 dpc blastocysts cultured in control L cells CM, Wnt5a CM and Wnt7a CM for varying incubation times. Binding activity was visualized in green, and nuclei were stained with iodure propidium (red). (**B**) Fluorescent intensity of the green signal was quantified and used to evaluate the binding activity of 3.5 dpc blastocysts cultured in control L cells CM, Wnt5a CM and Wnt7a CM for varying incubation times. * 2 way ANOVA test, P < 0.05 and Šídák’s multiple comparisons test. Different letters indicate a significant difference, while the same letter indicates a non-significant difference

Integrins expressed at the surface of the trophectoderm are known to interact with fibronectins present in the extracellular matrix of the uterus to allow adhesion of the blastocyst to the uterine luminal epithelium [8]. To evaluate the adhesive activity of integrins, FN-binding assays were performed on blastocysts treated for varying incubation times with either 10% control CM from L cells, 10% Wnt5a CM and 10% Wnt7a CM. A FN-binding assay involves incubating blastocysts with fluorescent polystyrene microspheres coated with fibronectin and assessing their binding capacity by measuring the fluorescent intensity of the bound microspheres. Since the addition of 10% CM from L cells did not affect Itgα5β1 expression or localization, this condition was selected as control for the following experiments. Weak FN-binding at the surface of the mural trophectoderm cells was detected in blastocysts incubated with control CM from L cells and the signal intensity appeared similar for all timepoints (Fig. 2A). In contrast, addition of Wnt5a or Wnt7a CM resulted in an increase in FN-binding activity after a 5 min incubation and a stronger signal was detected after a 10 min incubation (Fig. 2A). Quantification of the FN-binding activity was assessed by measuring the fluorescent intensity of the bound microspheres. Although no significant increase in FN-binding activity was observed in control CM, addition of Wnt5a or Wnt7a CM resulted in a statistically significant increase in FN-binding activity after a 10 min incubation (Fig. 2B). These findings demonstrated that a short exposure of blastocysts to Wnt5a or Wnt7a CM is sufficient for integrin translocation to the apical surface of the mural trophectoderm resulting in an increased FN-binding activity.

### Wnts present in the uterus are responsible for increased FN-binding activity

**Fig. 3:**
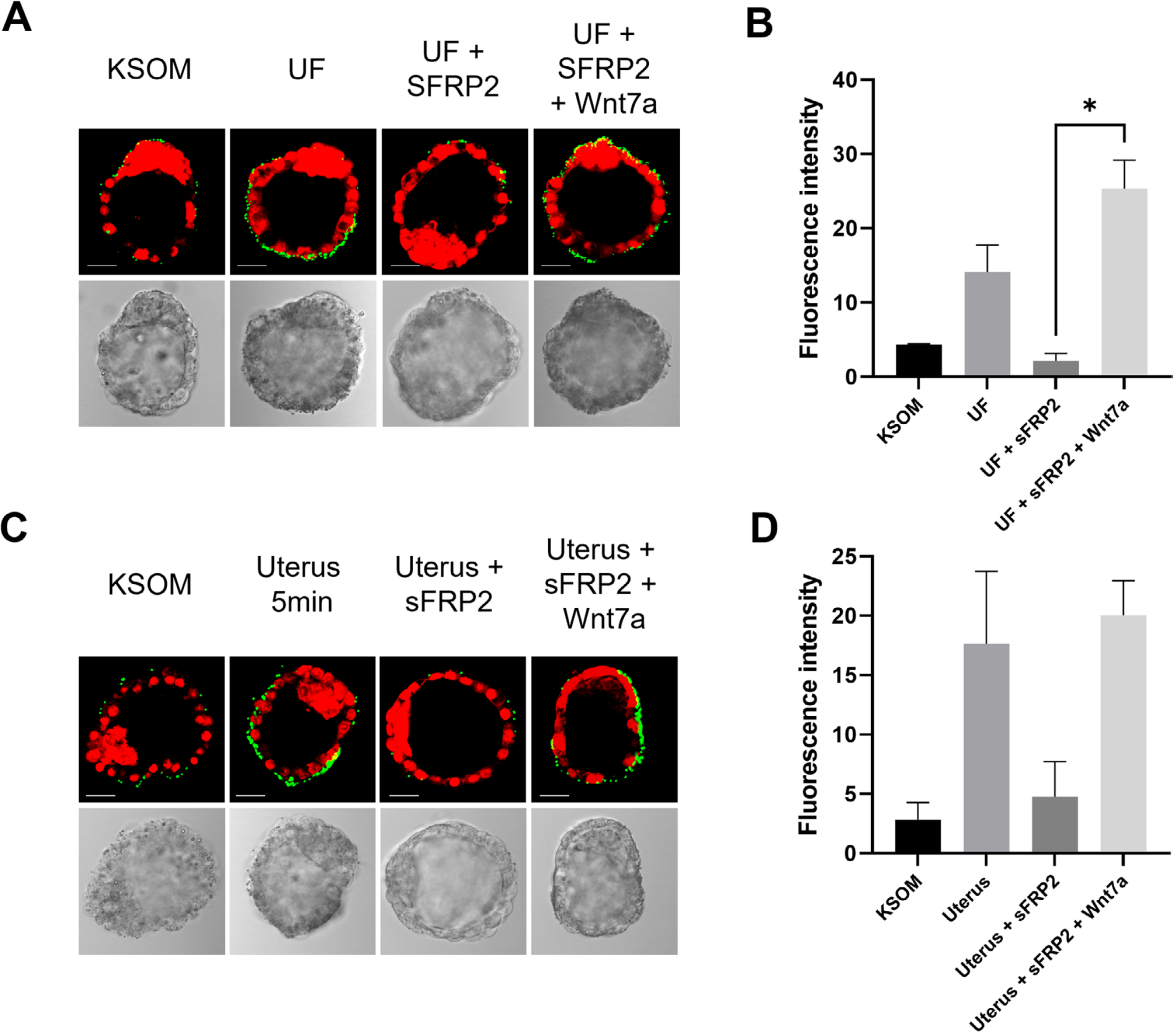
FN-Binding assays of peri-implantation blastocysts in vitro and in vivo. (**A**) Microscopy images of FN-binding assays realized on 3.5 dpc blastocysts cultured in KSOM alone, uterin fluid (UF) ± sFRP2 ± Wnt7a CM. Binding activity was visualized in green and nuclei were stained with iodure propidium (red). (**B**) Fluorescent intensity of the green signal was quantified and used to evaluate the binding activity of 3.5 dpc blastocysts cultured in KSOM alone, uterin fluid (UF) ± sFRP2 ± Wnt7a CM. * ANOVA test, P < 0.05 and Holm-Šídák’s multiple comparisons test. (**C**) Microscopy images of FN-binding assays realized on 3.5 dpc blastocysts cultured in vitro in KSOM or transplanted into the uterus with KSOM ± sFRP2 ± Wnt7a CM. Binding activity was visualized in green, and nuclei were stained with iodure propidium (red). (**D**) Fluorescent intensity of the green signal was quantified and used to evaluate the binding activity of 3.5 dpc blastocysts in vitro in KSOM or transplanted into the uterus with KSOM ± sFRP2 ± Wnt7a CM. * ANOVA test, P < 0.05 and Holm-Šídák’s multiple comparisons test

Several factors that regulate blastocyst development and implantation competence are derived from the uterus [17, 19–21]. To evaluate if uterine Wnt proteins are one of the factors responsible for regulating FN-binding activity of the mural trophectoderm, we tested whether uterine fluid could induce this FN-binding activity. Hatched blastocysts were incubated for one hour in vitro either with KSOM alone or KSOM with the addition of uterine fluid collected from 4.0 dpc pseudopregnant mice. Quantification of fluorescent intensity showed that blastocysts incubated with uterine fluid had an increase in FN-binding activity (fluorescence intensity 14.15 ± 6.24) compared to the those incubated with KSOM alone (fluorescence intensity 4.33 ± 0.17) (Fig. 3A and B). Interestingly, addition of sFRP2, a Wnt inhibitor to uterine fluid induced a decrease of FN-activity (fluorescence intensity 2.15 ± 1.74) and this could be rescued by the addition of excess Wnt7a CM (fluorescence intensity 25.33 ± 6.68) (Fig. 3A and B).

To further demonstrate that uterine fluid-derived Wnts were the in vivo factor responsible for the increase in FN-binding activity, hatched blastocysts were transferred for 5 min into the 4.5 dpc pseudopregnant mice uterus alone or in uteri that had been injected with sFRP2 30 min prior to blastocyst transfer. The presence of the blastocyst for 5 min within the uterus was sufficient to increase the FN-binding activity (fluorescent intensity 18.01 ± 6.13) compared to KSOM incubated blastocysts (fluorescent intensity 2.28 ± 1.72) (Fig. 3C and D). Addition of sFRP2 before blastocysts transfer resulted in a decrease in FN-binding activity (fluorescent intensity 4.77 ± 4.14) which could be rescued when Wnt7a was injected with sFRP2 before blastocyst transfer (fluorescent intensity 20.03 ± 4.13) (Fig. 3C and D). These results are evidence for the role of Wnts proteins found in the uterine fluid in the increase of FN-binding activity of the trophectoderm.

### Wnts signal through CaMKII to modulate FN-binding activity

**Fig. 4:**
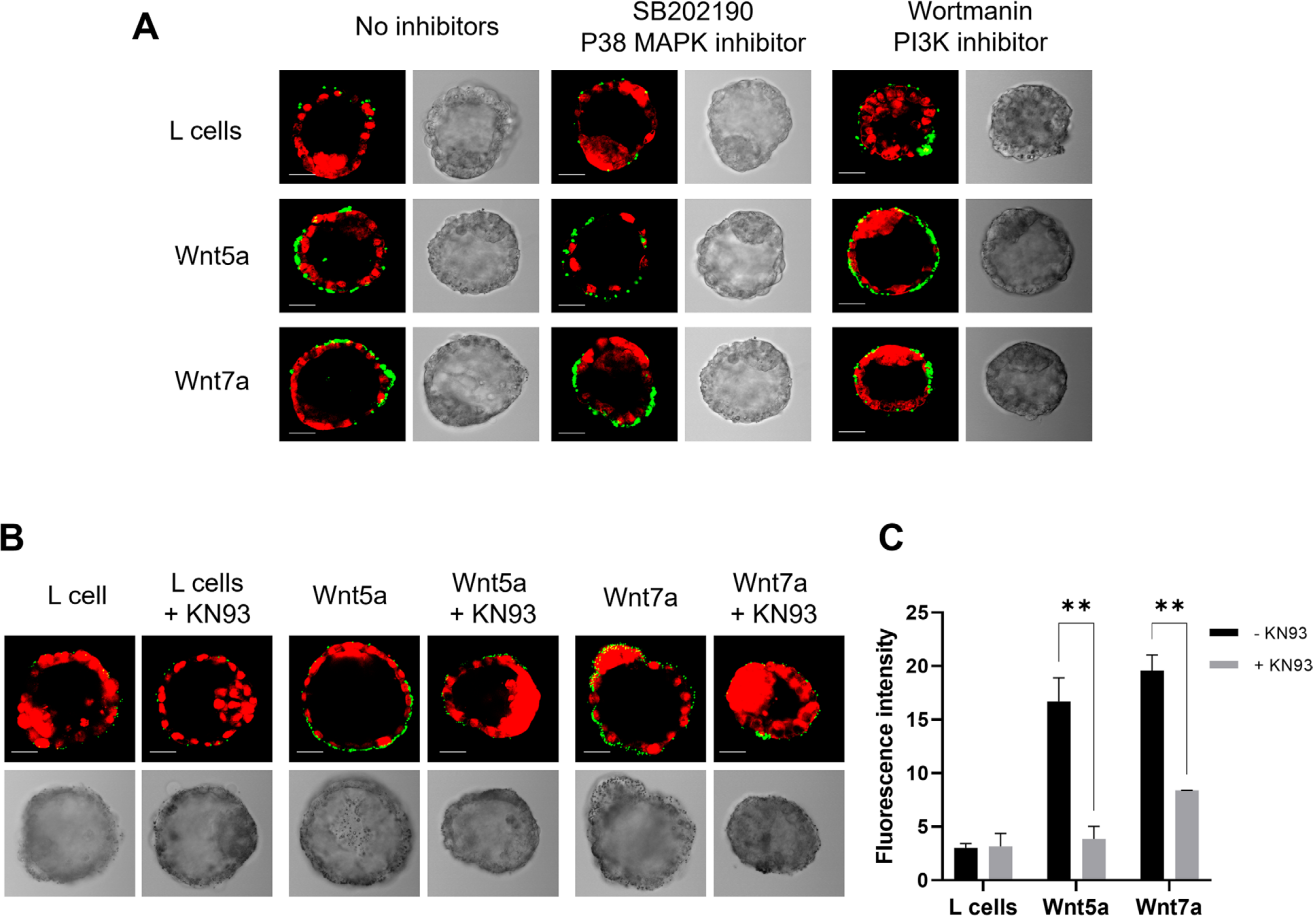
FN-Binding assays of peri-implantation blastocysts treated with Wnt proteins and different inhibitors. (**A**) Microscopy images of FN-binding assays realized on 3.5 dpc blastocysts cultured in control L cells CM, Wnt5a CM and Wnt7a CM with the addition of different inhibitors. Binding activity was visualized in green, and nuclei were stained with iodure propidium (red). (**B**) Microscopy images of FN-binding assays realized on 3.5 dpc blastocysts cultured in control L cells CM, Wnt5a CM and Wnt7a CM and with the CamKII inhibitor KN93. (**C**) Fluorescent intensity of the green signal was quantified and used to evaluate the binding activity of 3.5 dpc blastocysts cultured in control L cells CM, Wnt5a CM and Wnt7a CM and with the CamKII inhibitor KN93. **2way ANOVA, P < 0.01 and Dunnett’s multiple comparisons test

Wnt signalling is a highly complex signaling network with considerable overlap between different pathways. Some of these pathways, such as the active MAPK and PI3K pathways, have been shown to be important for blastocyst differentiation and regulation [28, 31–35]. Before determining the intracellular signaling pathway by which Wnt acts, we first tested the potential involvement of the MAPK and PI3K pathways. Inhibitors of p38 MAPK and PI3K were added to the blastocyst culture medium to determine if the activation of these pathways were necessary to observe the effects of Wnt proteins on FN-binding activity. Immunofluorescence showed, in the absence of inhibitors, an increase in the FN-binding activity after incubation with Wnt5a CM and Wnt7a CM compared with control CM, as previously shown (Figs. 2A and 4A). Addition of p38 MAPK inhibitor (SB202190) and PI3K inhibitor (Wortmanin) did not affect the ability of Wnt proteins to increase the FN-binding activity of blastocyst (Fig. 4A). These results suggested that MAPK and PI3K pathways are not required for the mechanism of Wnt proteins to increase FN-binding activity.

The Wnt signaling pathway has previously been shown to signal through the canonical b-catenin pathway (Wnt/b-catenin) and two non-canonical pathways, the Planar Cell Polarity (Wnt/PCP) and the Calcium pathway (Wnt/Ca^2+^) [36]. We have previously demonstrated that the Wnt/b-catenin pathway is not active in the blastocyst [30]. Furthermore, inhibition of two of the Wnt/PCP intracellular signaling components, JNK (SP600125) and ROCK (Y27632) had no effect on the increase of FN-binding activity in response to Wnts (data not shown). Since the increase of intracellular Ca^2+^ level had previously been shown to accelerate the trafficking of Itgα5β1 to the plasma membrane [21], we tested the involvement of the Wnt/Ca^2+^ pathway. This pathway involves activation of calcium/calmodulin-dependent protein kinase II (CamKII) in several tissues and organisms [37, 38]. CamKII is Ca^2+^/calmodulin-activated dodecameric enzyme involved in Ca^2+^ signaling. To determine whether Wnt proteins regulated the FN-binding activity of blastocysts by this mechanism, blastocysts were incubated with Wnt5a CM and Wnt7a CM alone or with the addition of the CamKII inhibitor KN93. Immunofluorescence staining showed that in the absence of inhibitors there was an increase of FN-binding activity after incubation with Wnt5a CM and Wnt7a CM compared to the control CM (Fig. 4B and C). Strikingly, the addition of CamK II inhibitor abolished the ability of Wnt proteins to induce FN binding activity (Fig. 4B). Quantification confirmed a significant decrease of the FN-binding activity of blastocysts treated with KN93 (control L cell CM 3.15 ± 1.7; Wnt5a CM 3.86 ± 1.63; Wnt7a CM 8.40 ± 0.01) compared to the ones treated only with Wnt5a and Wnt7a CM medium (control L cell CM 3.04 ± 0.56; Wnt5a CM 16.70 ± 3.10; Wnt7a CM 19.58 ± 2.04) (Fig. 4C). This result demonstrated that Wnt proteins signal through CamKII of the Wnt/Ca^2+^ non-canonical intracellular signaling pathway to regulate the FN-binding activity of blastocysts.

### Wnt regulation of FN-binding activity is required for blastocyst attachment to the luminal epithelium

**Fig. 5:**
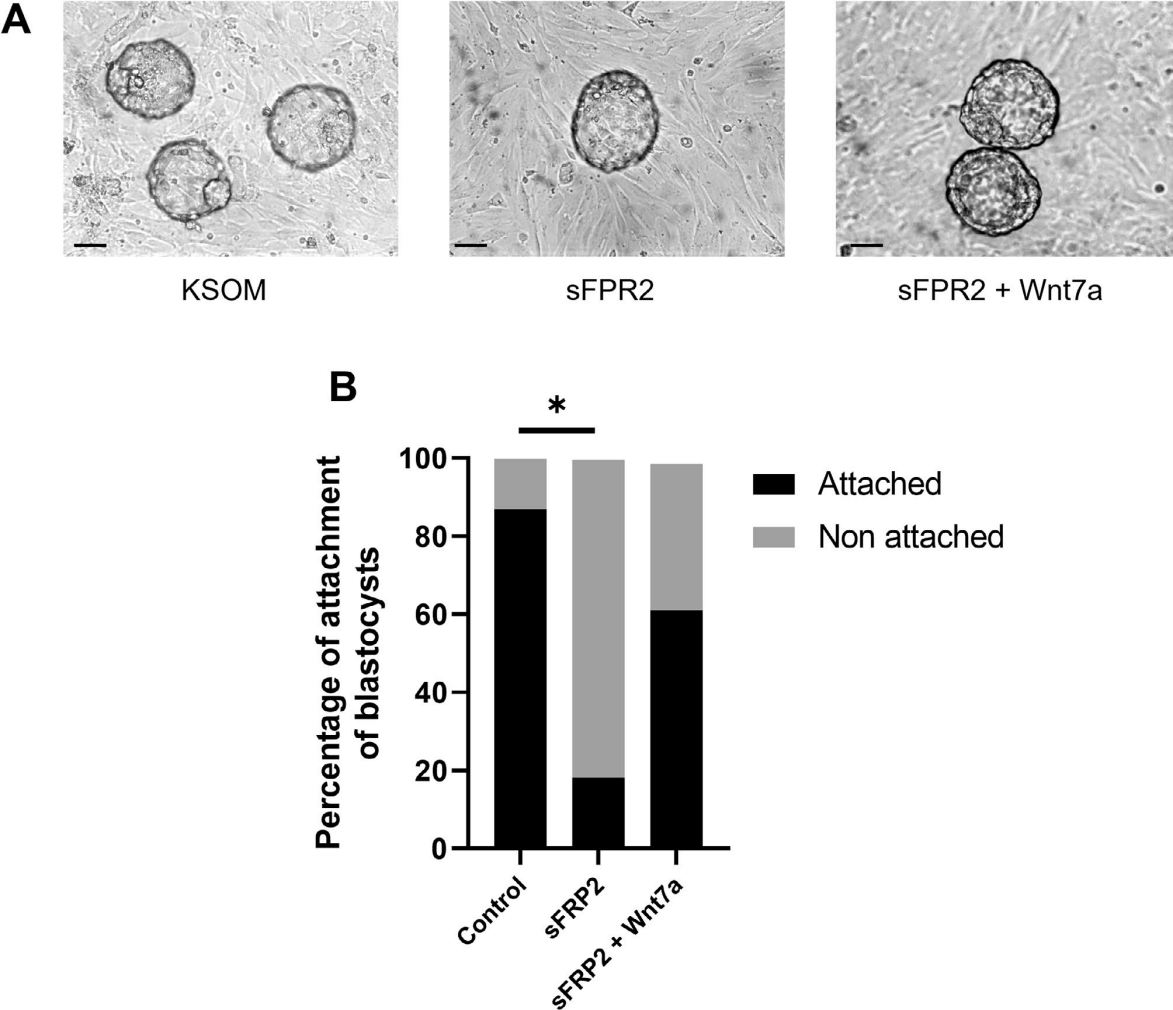
Attachment assays between peri-implantation blastocysts and luminal epithelial uterine cells. (**A**) Bright-field photomicrographs of co-culture between hatched blastocysts and luminal epithelial uterine cells in KSOM alone, with sFRP2 or with sFRP2 and Wnt7a CM. (**B**) After 2 h of incubation, attachment of blastocysts to the epithelial cells were quantified by moving the plates and counting the number of blastocysts attached or non attached. * ANOVA test, P < 0.05 and Holm-Šídák’s multiple comparisons test

Since attachment of the blastocyst to the uterine epithelium is an important step in implantation, we wanted to evaluate the functionality of blastocysts in vitro. Co-culture experiments were performed between primary luminal epithelial cells plated on collagen and hatched blastocysts in a medium composed of KSOM alone, KSOM with SFRP2 or with SFRP2 and Wnt7a CM. After 2 h of co-culture, 86.90 ± 9.69% of blastocysts cultured in KSOM alone were attached to the cells while 18 ± 7.35% of blastocysts cultured in KSOM plus sFRP2 were attached. Addition of Wnt7a CM to sFRP2 partially restored the attachment rate of blastocysts with 63 ± 17.68% of them attached (Fig. 5). This result confirmed that Wnts proteins have a functional effect on blastocyst attachment to uterine epithelial cells.

## Discussion

In this study we demonstrated a novel role for the Wnt proteins in blastocyst adhesion to uterine epithelial cells. More specifically, we showed that Wnt5a, Wnt7a and uterine fluid were able to induce the Wnt/Ca^2+^ non-canonical pathway via activation of CamKII. This mechanism induced the migration of Itgα5β1 to the apical pole of the blastocyst resulting in increased FN-binding activity of the mural trophectoderm.

The presence of several integrins in the peri-implantation blastocyst has been described previously [12, 13]. After treatment with Wnt5a and Wnt7a proteins, the total amount of Itgα5β1 in the trophectoderm was not affected while an increase of the Itgα5β1 on the surface was observed, suggesting that Wnt proteins induced a trafficking of integrin from the basal to the apical surface of trophectoderm. This result is consistent with previous studies that have shown that the main regulation of integrin in the late blastocyst is through trafficking rather than gene regulation or protein synthesis [7, 18]. The addition of Wnt5a and Wnt7a CM also resulted in a significant increase of the FN-activity and correlated with the early migration of the Itgα5β1 to the apical domain of the mural trophectoderm. However, whereas treatment with Wnt5a and Wnt7a induced a non-significant trend toward increased surface staining of Itgα5β1, they induced a significant increase in FN-binding activity. Since Itgα5β1 is not the only integrin involved in blastocyst adhesion with fibronectin, this suggests that Wnt proteins may also impact other integrins, leading to a greater increase in FN-binding [13]. For example, it has been described that HB-EGF acts on two different integrins, resulting in a migration of ItgαVβ3 and Itgα5β1 to the apical pole of the blastocyst [21, 39].

Several factors which are produced by the uterus during the peri-implantation period have been shown to be capable of modulating trophoblast adhesion [16]. Our results demonstrated that 3.5 dpc blastocysts cultured for 30 min in uterine fluid or cultured for 3 days in vitro and transferred for 5 min into the 4.5 dpc pseudopregnant mouse uterus showed a higher FN-binding activity compared to their respective controls confirming that the uterus provides an optimal environment for the adhesion development of the blastocyst. To target the effect of Wnt proteins in our experiments, we used sFRP2, a Wnt receptor antagonist, to inhibit the effect of endogenous Wnt proteins present in the uterus or the uterine fluid, and then added Wnt7a CM in addition to sFRP2. The results that we obtained confirmed that the increased FN-binding activity was initiated by the actions of uterine fluid-derived Wnt proteins. We have previously demonstrated that Wnt proteins from the blastocysts were able to activate Wnt/β-catenin pathway in the uterus [22]. Indeed, transfer of beads coated with Wnt7a in uterine horns of pseudopregnant TCF/Lef-LacZ reporter female mice on the morning of day 4 induced Wnt/β-catenin signaling activity in a banding pattern along the uterine horns. Wnt proteins thus seem to have a dual role by acting on the blastocyst to regulate the integrin trafficking but also on the uterus to activate the Wnt/β-catenin signaling during the peri implantation period. In our study, we showed that transferring of blastocysts along with sFRP2 into the uteri of pseudopregnant females resulted in a significant decrease in blastocyst implantation as compared to controls [22]. Similarly, injection of sFRP2 to naturally mated female on day 4 leads to significant reduction of implantation. These results correlate with the decrease of blastocyst attachment that we observed with the addition of sFRP2 in our co-culture of blastocysts and luminal epithelial cells. These two studies taken together demonstrates that the inhibition of the action of Wnt proteins leads to decreased implantation both in vitro and in vivo confirming the requirement of Wnts in successful embryo adhesion and subsequent implantation.

Effects of Wnt signaling, or more specifically Wnt5a and Wnt7a proteins, on the implantation stages of blastocysts have already been described in vitro using cell lines [40–43]. A recent study using Jeg-3 spheroids as a human trophoblast surrogate demonstrated that Wnt signaling increased spheroid attachment and outgrowth [43]. Studies on the human extravillous trophoblast line SGHPL-4 and HTR-8/SVneo showed that Wnt5a protein increased migration and invasion of trophoblast using a transwell assays while Wnt5a knockdown significantly suppressed invasion of the cells [41, 42]. Wnt5a could also be a critical regulator of proliferation and survival of human trophoblast cells [35]. Additionally, significantly lower WNT5A mRNA levels were observed in a cohort of 43 patients with recurrent implantation failure compared to 71 control women [44, 45].

Since the increase of FN-binding activity that we observed after treatment with Wnt proteins is also known to occur upon elevation of intracellular Ca^2+^, and some Wnts proteins stimulate intracellular Ca^2+^ release and activate two kinases, CamKII and PKC through a non-canonical pathway, we wanted to evaluate the mechanism of action of Wnt5a and Wnt7a proteins [19, 21, 46]. We demonstrated that Wnt protein’s mechanism of action needs activation of CamKII to allow the trafficking of integrin protein and FN-binding activity of the blastocyst. Wnt5a and Wnt7a were already described to be able to initiate non-canonical Wnt signaling [47, 48]. These results also support other studies to suggest that intracellular Ca^2+^ flux induces activation of CaMKII which is essential for the increase of FN-binding activity [38]. A study from 2003 also showed that integrin stimulation by FN generates an auxiliary calcium signal that activates calmodulin and CaMKII with the formation of a Raf-1/CaMKII complex [49]. Moreover, a study from 2019 revealed that KN-93 binds directly to Ca(2+)/CaM and not to CaMKII, and the inhibition effect that we saw using this inhibitor on the FN-binding activity induced by Wnt proteins confirmed the central role of Ca^2+^ and calmodulin in the mechanism of action of Wnt proteins [50]. However, a study using embryo transfer experiments revealed that silencing of the Wnt-β-catenin pathway in the blastocyst altered the blastocysts’ competency to implant suggesting that both Ca^2+^ non-canonical and canonical pathways are important for the implantation of the blastocyst [26].

In conclusion, our findings demonstrate that Wnt proteins from the uterine fluid induces the trafficking of the Itgα5β1 into the surface of the blastocyst which allows the ligation of integrin to fibronectin and the attachment of the blastocyst to the endometrium. Our results thus uncovered a novel role for Wnt signaling in the acquisition of an adhesion-competent blastocyst state prior to implantation.

## Data Availability

The datasets used and/or analysed during the current study are available from the corresponding author on reasonable request.
